# Delay in weight bearing in surgically treated tibial shaft fractures is associated with impaired healing: a cohort analysis of 166 tibial fractures

**DOI:** 10.1007/s00590-018-2190-2

**Published:** 2018-04-09

**Authors:** I. B. Houben, M. Raaben, M. Van Basten Batenburg, T. J. Blokhuis

**Affiliations:** 1 Department of Surgery, University Medical Centre Utrecht, Heidelberglaan 100, 3584 CX Utrecht, The Netherlands; 2 Department of Surgery, Maastricht University Medical Centre, P. Debyelaan 25, 6229 HX Maastricht, The Netherlands

**Keywords:** Tibia, Fracture, Weight bearing, Intramedullary nail, Fracture healing

## Abstract

**Background:**

The relation between timing of weight bearing after a fracture and the healing outcome is yet to be established, thereby limiting the implementation of a possibly beneficial effect for our patients. The current study was undertaken to determine the effect of timing of weight bearing after a surgically treated tibial shaft fracture.

**Materials and methods:**

Surgically treated diaphyseal tibial fractures were retrospectively studied between 2007 and 2015. The timing of initial weight bearing (IWB) was analysed as a predictor for impaired healing in a multivariate regression.

**Results:**

Totally, 166 diaphyseal tibial fractures were included, 86 cases with impaired healing and 80 with normal healing. The mean age was 38.7 years (range 16–89). The mean time until IWB was significantly shorter in the normal fracture healing group (2.6 vs 7.4 weeks, *p* < 0.001). Correlation analysis yielded four possible confounders: infection requiring surgical intervention, fracture type, fasciotomy and open fractures. Logistic regression identified IWB as an independent predictor for impaired healing with an odds ratio of 1.13 per week delay (95% CI 1.03–1.25).

**Conclusions:**

Delay in initial weight bearing is independently associated with impaired fracture healing in surgically treated tibial shaft fractures. Unlike other factors such as fracture type or soft tissue condition, early resumption of weight bearing can be influenced by the treating physician and this factor therefore has a direct clinical relevance. This study indicates that early resumption of weight bearing should be the treatment goal in fracture fixation.

**Level of evidence:**

3b.

## Introduction

Impaired healing of tibial fractures remains a challenge in orthopaedic surgery, affecting quality of life and posing economic problems [1, 2]. A sophisticated approach in management and stimulation of the bone healing process is necessary to overcome or avoid these problems. The so-called Diamond Concept describes five factors contributing to bone healing: osteogenic cells, osteoinductive stimulants, osteoconductive scaffolds, vascularity and mechanical environment [3]. The first four of these are mainly patient related and reflect the biological activity of the patient. In contrast, the mechanical environment is under the direct influence of the treating physician, since the mechanical environment is the direct result of treatment options (i.e. a cast or a plate) and decision-making (i.e. weight bearing or not). The relevance from a proper mechanical environment finds its origin in several key cellular processes that contribute to bone healing. After activation by external mechanical loading, mechanosensory osteocytes modulate the recruitment and activity of osteoblasts and osteoclasts, leading to remodelling of bone and bone formation through a process referred to as mechanotransduction. The mechanisms of mechanotransduction have been widely explored in in vitro studies [4]. It is known from animal studies that immediate high strain rates, comparable to walking strains, induce a greater amount of callus formation during the early phase of fracture healing [5–7]. Others have shown a dose-dependent stimulation of enchondral ossification by interfragmentary strains [8]. Despite these studies, translation of the concept of mechanotransduction to clinical practice has not yet been achieved. A direct relation between mechanical loading and healing outcome has not yet been established.

Current concepts in fracture fixation suggest that micromovement in the fracture should take place and that early weight bearing should be initiated as soon as possible after fracture management [9]. A common argument against early weight bearing is possible loss of reduction. However, a recent biomechanical study has shown that angular stable locking plates may allow full or at least partial immediate weight bearing [10]. A recent randomized controlled trial has shown the safety of early weight bearing after intramedullary fixation [11]. Moreover, withholding weight bearing causes loss of bone density. Not loading the bone for 8 weeks after a fracture reduces bone density up to 1 year after the fracture [12]. Additionally, prolonged inactivity quickly affects skeletal muscle tissue [13], leading to decreased mechanosensory function, atrophy and even increased energy expenditure on ambulation [14]. The aim of this study was to explore the relation between timing of weight bearing and fracture healing after tibial shaft fracture surgery. We hypothesized a positive correlation between early initial weight bearing and fracture healing outcome, suggesting that delay of initial weight bearing will increase the rate of impaired healing. In order to assess the safety of weight bearing, the fixation failure rate was also analysed.

## Materials and methods

All consecutive tibial shaft fractures surgically treated in a level-I trauma centre between January 2007 and March 2015 were analysed. Data were retrieved from electronic medical records. Only surgically treated diaphyseal fractures of the tibia were included according to the AO/OTA rule of squares described by Müller et al. [15]. All patients with impaired fracture healing (IFH) were identified. IFH was defined as fractures without consolidation (see below) with a minimum duration of at least 6 months after the fracture. In this manner, patients with a delayed union as well as patients with a non-union as defined by the Food and Drug Administration were included [16]. Early radiographic follow-up consisted of an intraoperative or early post-operative tibial multiplane radiograph. Further follow-up radiographic imaging was performed within 6 months and a second time after 12 months post-operatively. Thereafter, follow-up was continued in clinical or radiological impaired healing cases. Patients aged under 16 years at the time of injury and fractures followed by an amputation within 9 months after injury were excluded from this study. If no clinical follow-up was performed at 9 months, patients with IFH were excluded.

Initial weight bearing (IWB) was defined as the first documented moment the patient was able to load the affected leg with any weight exceeding touch-down weight bearing (TDWB), which in earlier studies is defined as loading between 15 and 35 lbs. (6.82–15.91 kg) [17, 18]. Healing time was defined as the time between injury and pain-free full weight bearing with radiological signs of progressive consolidation. Radiological union was assessed using radiographs in two orthogonal planes, commonly anteroposterior views and lateral views. When three out of four cortices demonstrated cortical bridging or complete disappearance of fracture lines, radiological union was scored [19]. Additionally, for the non-unions and for delayed unions the healing type was assessed independently by two of the investigators (MR and TB). The radiographs were classified as atrophic or hypertrophic bone healing. Clinical healing was defined as the ability to bear full weight without pain [20].

Data on body mass index (BMI), injury severity score (ISS), smoking, non-steroidal anti-inflammatory drugs (NSAID) use, steroid use, neurological trauma, presence of other orthopaedic injuries, infections requiring surgery, fixation treatment and reaming or non-reaming in IM nailing surgery were retrieved from medical records. Since previous studies have identified fracture type, fasciotomy and open fractures as clear risk factors for impaired healing [21–24], these risk factors were also included in the analysis. Smoking was scored positive if patients smoked during fracture healing or within 6 months before trauma, all regardless of quantity. Two drug types were included in order to assess drug use influencing fracture healing: NSAIDs and steroids [25]. The majority of patients received NSAIDs post-operatively for three to 7 days. NSAIDs taken over a longer period than 1 week post-operatively were assessed as drug use influencing fracture healing [26]. Both NSAIDs and steroids were assessed regardless of frequency and quantity. Fracture classification was assessed according to the AO/OTA classification. In order to maintain a reliable assessment, only the main categories type A, B or C, representing, respectively, simple, wedged and complex fractures were used [27]. Open fractures were assessed according to the Gustilo classification [28]. Traumatic brain injury (TBI) was categorized according to the Glasgow Coma Scale (GCS), where a GCS of 13–15 was classified as mild TBI, a GCS of 9–12 as moderate, and a GCS of 3–8 as severe [29]. Mild TBI was considered as TBI not affecting rehabilitation, and moderate or severe TBI was registered as neurological trauma affecting rehabilitation and was therefore included in the analysis. Furthermore, tibial fracture patients with vertebral injury, pelvic injury, ipsilateral lower extremity injury or contralateral lower extremity injury were documented. This was done in order to assess whether other orthopaedic injuries possibly preventing rehabilitation increased the chance of developing IFH.

Infections requiring surgery (infection procedures) that occurred within 9 months after trauma were included in the primary analysis as a possible risk factor for impaired healing outcome [30]. The following procedures were considered infection surgeries: placement of gentamicin beads, antibiotic-coated nail placement, sequestrectomy, total removal of implant material, or debridement. Finally, fixation failure was assessed and comprised both implant failure (assessed in case of radiologic reported bending or breakage of the main implant, including the screws) and loss of reduction with loosening of the implant.

### Statistical analysis

All data are shown as mean ± standard deviation (SD), unless otherwise specified. Data were analysed in four steps. First, data distribution was assessed. Data on BMI and ISS and IWB were all positively skewed, and therefore a logarithmic function of these variables was used for further analysis. Data on age were moderately positively skewed, and therefore a square root transformation was calculated and used. Second, the correlation between our predictor (initial weight bearing) and our outcome variable (IFH) was assessed. Third, the role of possible confounders in the relation with both the predictor and the outcome variable was examined using separate bivariate correlation tests. Three evident literature-based risk factors for developing IFH were used in the regression analyses (see final step) as possible confounders, regardless of their outcome during correlation tests. These were fracture type, fasciotomy and open fractures [21–24]. As a final step, regression analysis was performed to test the predictive value of IWB on the healing outcome in a multivariate analysis. All possible confounders were stepwise added (forward) and dropped (backward) from the regression model to see whether the predictive value of our predictor (IWB) was influenced. All tests were two-sided with a significance set at *p* < 0.05. Data were analysed using SPSS version 23.0 (IBM SPSS Statistics for Windows, Version 23.0, Armonk, NY).

## Results

**Fig. 1:**
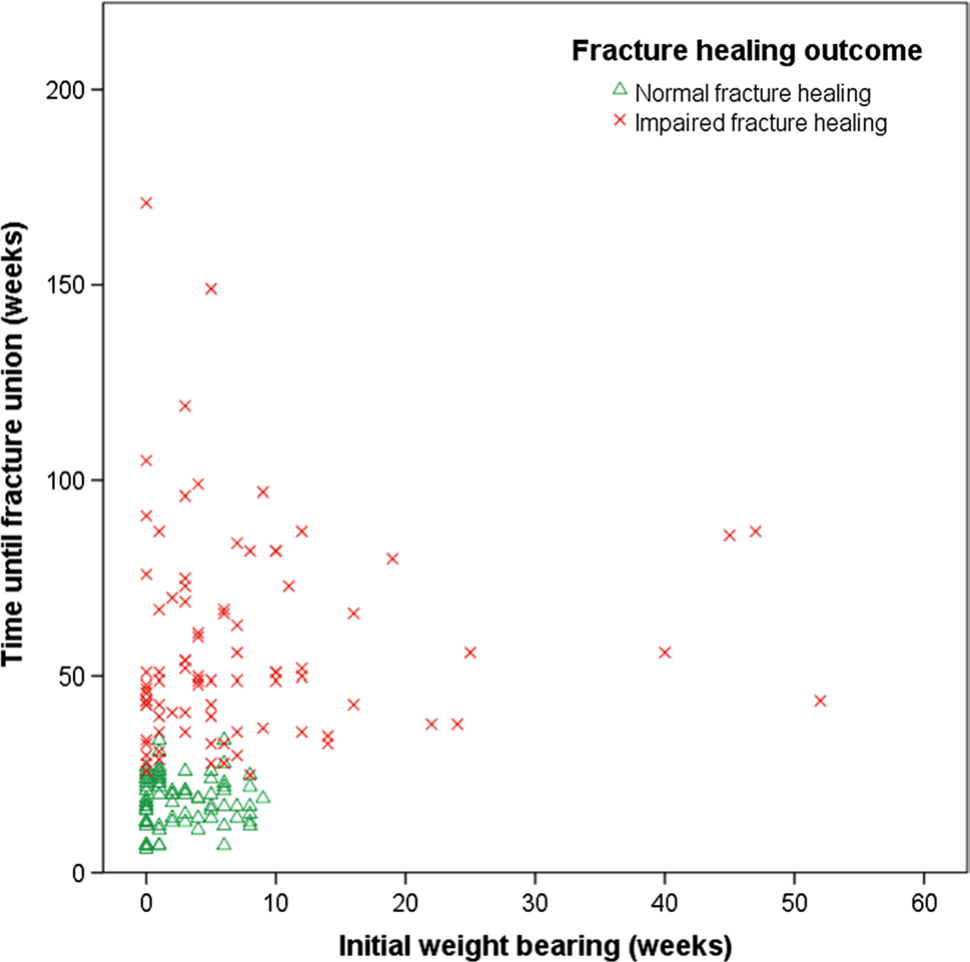
A scatter plot with the timing of initial weight bearing (IWB) in weeks on the *x*-axis and weeks until fracture union after trauma on the *y*-axis. The plot is clustered for fracture healing outcome. The green triangles represent normal fracture healing, and the red crosses represent impaired fracture healing (color figure online)

In 1200 patients, tibial fracture in one or both limbs was diagnosed, including all diaphyseal and metaphyseal fractures. Totally, 608 of these were tibial shaft fractures. Following our exclusion criteria, a total number of 166 surgically treated diaphyseal tibial fractures were treated in 161 patients. Impaired fracture healing (IFH) occurred in 86 fractures and 80 fractures healed uneventful (normal fracture healing, NFH). Of the impaired fractures, 41 were delayed unions and 45 were non-unions. Age, gender, smoking status and drug use were similar in both healing groups (Table 1). Data on smoking status and drug use were documented in 116 and 151 cases, respectively. In total, 93 fractures were open and 73 were closed. Of the open fractures, 58 occurred in the impaired healing group. Twenty-eight fractures in the impaired healing group were closed. All demographic data are summarized in Table 1. Evident differences in weight bearing were seen between the NFH group and the IFH group (Fig. 1). Initial weight bearing was significantly later in the IFH group (7.4 weeks ± SD 10.3) than in the NFH group (2.6 weeks ± SD 2.8) *R* = 0.301, *p* < 0.001 (Table 2). Among the non-union patients, 18 (40%) had an atrophic type of non-union. This was similar to the delayed union group (17 patients, 41%). For the non-union group, initial weight bearing showed no significant correlation with the type of non-union (*p* = 0.892). Between both delayed and non-union groups and within the groups, no significant differences were found between smokers and healing type (lowest *p* value = 0.384).

**Table 1:** Demographics, smoking status and drug use of cases with normal (within 6 months) or impaired diaphyseal tibial fracture healing

Patient characteristics	Normal healing (*n* = 80)	Impaired healing (*n* = 86)	Bivariate *p* value IWB	Bivariate *p* value healing outcome
Age, mean years (± SD)	36.8 (19.0)	40.5 (17.9)	0.601	0.146
Gender male; female	51; 26	66; 20	0.931	0.095
BMI, median (interquartile range)^a^	23.4 (21.2–25.1)	25.8 (23.4–27.8)	0.323	*0.010*
Smoking status			0.514	0.295
Smoking	23	24		
Non-smoking	27	42		
Unknown	30	20		
Drug use			0.746	0.206
NSAIDs	7	14		
Steroids	2	2		
Both	–	2		
No drug use	65	59		
Unknown	6	9		

Correlation analysis with initial weight bearing (IWB) and with healing outcome was done for each variable. Significant correlation is marked in italic font

^a^59 cases had missing data

**Table 2:** Trauma, fracture and management characteristics of cases with normal (within 6 months) or impaired diaphyseal tibial fracture healing

Patient characteristics	Normal healing (*n* = 80)	Impaired healing (*n* = 86)	Bivariate *p* value IWB	Bivariate *p* value healing outcome
ISS, mean	14.8	15.4	0.440	0.844
AO/OTA type of fracture			*0.001*	< *0.001*
Simple	44	23		
Wedge	29	26		
Complex	7	37		
Open fracture	35	58	0.168	*0.002*
Other orthopaedic injuries affecting rehabilitation			*0.006*	0.213
Ipsilateral lower extremity fracture	7	2		
Both lower extremities fractured	7	15		
Pelvic injury	1	2		
Vertebral injury	2	5		
Polytrauma (> 2 of above-named injuries)	5	5		
Neurological trauma affecting rehabilitation	9	10	0.408	0.939
Treatment			< *0.001*	0.080
Intramedullary nailing	76	75		
Open reduction internal fixation	4	11		
IM nails reamed (%)^a^	38.9	56.6	0.329	0.067
Fasciotomy	5	27	0.070	< *0.001*
Infection procedures	1	17	*0.009*	< *0.001*
Duration of fracture healing, mean in weeks (± SD)	18.4 (6.5)	57.1 (26.5)	< *0.001*	–
Timing of IWB, mean in weeks (± SD)	2.6 (2.8)	7.4 (10.3)	–	< *0.001*

Correlation analysis with initial weight bearing (IWB) and with healing outcome was done for each variable. Significant correlation is marked in italic font

^a^59 cases had missing data

Bivariate analysis identified a significant correlation of two variables with both initial weight bearing (in weeks) and IFH, being fracture type (*R* = 0.380, *p* < 0.001) and infection requiring surgical procedure (*R* = 0.298, *p* < 0.001). These variables were therefore identified as a possible confounder. *p* values on correlations with other variables as stated in the method section can be found in Table 2. Although the distribution of these factors was similar in our groups, fasciotomy and open fractures were already identified as possible confounders based on the literature (together with fracture type) [21–24]. In bivariate analysis, these two variables showed relatively high correlations with fracture healing (*R* = 0.319 and 0.239, respectively). Although no significance was found in correlations with both IWB and IFH outcome, they were included in the logistic regression model as possible confounders to isolate the relationship of initial weight bearing and IFH. Following a significant correlation after bivariate analysis (*R* = 0.298, *p* < 0.001), infection requiring surgery was also added to the model as a possible confounder. A logistic regression was performed to ascertain the effect of initial weight bearing on the likelihood of IFH occurring. The logistic regression model showed a significant relation between initial weight bearing and healing outcome (*χ*^2^(5) = 57.338, *p* < 0.001). The odds ratio (OR) of developing IFH was 1.13 per week delay in resumption of weight bearing (95% confidence interval 1.03–1.25). The model correctly classified 75.3% of cases and explained 39.0% (Nagelkerke R2) of the variance in fracture healing. Forward and backward stepwise regression showed that this relation was not influenced by any of the identified variables. Hence, while adjusted for four possible confounders, initial weight bearing significantly predicted IFH (*p* = 0.012). Regression coefficients and standard errors are shown in Table 3.

**Table 3:** Summary of multivariate logistic regression analysis with impaired fracture healing as our dependent outcome variable

	*p* value	Odds ratio	95% CI for OR	
			Lower	Upper
Initial weight bearing	0.012	1.13	1.03	1.25
Infection requiring procedure	0.143	5.23	0.571	48.01
Fracture type	0.002	2.19	1.34	3.58
Fasciotomy	0.040	3.39	1.06	10.84
Open fracture	0.109	1.84	0.87	3.87

Fixation failure occurred in sixteen cases. Thirteen of these sixteen fixation failures concerned screw breakage in IM nails. One of all sixteen failures concerned breakage of the nail, and one concerned breakage of the plate. Screw bending without breakage occurred in one patient.

In one patient, fixation failure occurred early after fracture fixation in a non-compliant patient. All other patients that showed fixation failure had started weight bearing at least 1 month prior to fixation failure. Mean time until fixation failure was 6 months (± SD 4.63 months) after injury (range 1–15.5 months including two outliers after 12 and 15.5 months).

Of all patients, 75 cases required additional surgery. Of these 75 cases, the number of additional procedures per case was 2.32 with a range of 1–11 procedures. Eighteen out of 75 patients required at least one additional procedure based on surgical infection management within 9 months after primary fixation (Table 2). These procedures were removal of osteosynthetics (4 patients), sequestrectomy (3 patients), deep cleaning and drainage of the fracture area (9 patients) and/or gentamicin beads or gentamicin-coated nail placement (7 patients). There were patients with infection having a combination of these procedures. Twenty-seven out of 75 cases received removal of screws for dynamization purposes to improve healing. In the remaining 20 cases, additional surgery consisted of superficial wound cleaning or removal of screws, because of skin damage or patient complaints.

## Discussion

Weight bearing after a fracture is important, as it helps to maintain bone and muscle mass and helps to return to daily life participation [13, 14]. Moreover, through a process referred to as mechanotransduction weight bearing supports bone healing. This positive effect, however, has been determined in laboratory-based studies mostly [5, 6, 8], and many surgeons are still hesitant to permit immediate weight bearing. This study showed a clear relation between healing outcome and initiation of weight bearing in 166 consecutive surgically treated tibial shaft fractures. In this cohort study, a significant correlation between timing of initial weight bearing and outcome in diaphyseal tibial fracture healing was identified. Patients who showed normal healing of their tibial fractures started initial weight bearing significantly sooner than patients who developed impaired healing. Moreover, in a multivariate analysis late resumption of weight bearing was identified as an independent risk factor for development of impaired healing. Although the odds ratio for the development of impaired healing is relatively small, it accounts for an odds ratio per week delay of weight bearing restriction, indicating a cumulative effect per week extra delay. Other risk factors are present and play an evident role, but in contrast to many other factors, weight bearing is under the direct influence of the treating physician.

Mechanical stability of the currently used osteosynthetic material allows for full weight bearing, and the average load supported by the injured limb in tibial fracture patients exceeds 50% of the uninjured limb at 1 week without any fixation failure [9, 10]. More recently, the safety of early weight bearing in tibial shaft fractures has been demonstrated in a prospective study on early weight bearing. Although fixation failure is often mentioned as an argument in postponing initial weight bearing, only sixteen patients (9.6%) of our total population developed a fixation failure. The majority consisted of inconsequently and cost-neutral bending or breakage of screws in IM nails, also referred to as autodynamization. In these cases, reoperation was not necessary. Considering the small number of clinically relevant fixation failures (1.8%) and the timing of failure (σ 6 months), fixation failure in this population is considered a result of impaired healing, instead of a cause. Safety is therefore not considered a valid argument against resumption of weight bearing in this population.

Given the retrospective design of our study, several limitations have to be kept in mind when interpreting these data. First, timing of initial weight bearing was retrieved from the electronic medical records. For each patient, initial weight bearing was defined as any loading exceeding touch-down weight bearing (TDWB) and the timing was identified based on notes of the doctor or the physical therapist. The physical therapists are all experienced and exclusively appointed to the trauma ward, but training of patients and documentation of progress are not necessarily performed every day. Thereby, underreporting of loading may play a role in the data. Since reporting is generally done more extensively in patients requiring more care, this effect is expected to be larger in the normal healing group. Second, as the definition of impaired fracture healing is still debated upon, delayed healing and non-unions were combined. This method may cause an overestimation of actual union healing complication, because many patients with delayed healing may have a seemingly natural healing course. Although this increases the heterogeneity of the impaired healing group, combining delayed and non-unions has increased the number of cases and resulted in increased analytical strength of the study. We realize that a non-union rate of 27% is high, compared to an average reported rate ranging from 5 to 17% [2, 11, 22, 30]. In contrast to the majority of trials, our series consisted of surgically treated tibial fractures encountered in a level-I trauma centre with relatively many open injuries and can therefore not be seen as representative for any general orthopaedic practice population. Moreover, this population has been injured mostly in high-energy trauma, which may be an independent risk factor for bone healing [31]. In our group, no difference in atrophic or hypertrophic healing pattern was seen in the impaired healing group compared to the non-union group, but the number of cases is relatively small to warrant a conclusion in this perspective. Third, the number of cases included in the final analysis is relatively small compared to the total number of patients treated during the study period. This is due to several factors, including treatment in a level-I facility, where patients are transferred to other institutions after initial stabilization and thereby are lost to follow-up. Finally, our data do not represent healing of all tibial fractures. Only surgically treated tibial shaft fractures were included. This has limited the sample size of our study and limits the external validity of our data for other fractures, such as non-operatively treated tibial shaft fractures. However, the homogeneity in our study group was increased by this inclusion, leading to improved rigidity of our conclusions.

Another consideration in interpretation of this study is the possibility of a reverse relationship between weight bearing and healing outcome; delayed healing may result in more pain, and therefore delay weight bearing, instead of delayed weight bearing leading to impaired healing. Two arguments are important in this perspective. First, the delay in weight bearing for both normal healing and impaired healing (2.6 and 7.4 weeks after surgery, respectively) is for both groups much shorter than the mean time to union, even for normal healing. In other words, delay in weight bearing is observed in the early phase of fracture healing, which is consistent with the concept of mechanotransduction. Second, healing outcome was defined as the dependent variable throughout the statistical evaluation. In both forward and backward logistic regression, the time of initial weight bearing was identified as an independent factor with a significant predictive value on healing outcome (*p* = 0.012). This rigorous statistical analysis indicates a clear effect of early weight bearing on positive healing outcome.

In conclusion, the current study is the first to describe the relation between delay in weight bearing and development of impaired healing of surgically treated tibial shaft fractures. A significant relation was observed (*p* = 0.012), indicating a risk of impaired healing that increases with each week in delay of weight bearing. The aim in treating tibial shaft fractures should therefore be to allow early weight bearing, exceeding touch-down weight bearing, as soon as possible.

## References

[1] Brinker MR, Hanus BD, Sen M, O’Connor DP (2013). The devastating effects of tibial nonunion on health-related quality of life. J Bone Joint Surg.

[2] Dahabreh Z, Calori GM, Kanakaris NK, Nikolaou VS, Giannoudis PV (2009). A cost analysis of treatment of tibial fracture nonunion by bone grafting or bone morphogenetic protein-7. Int Orthop.

[3] Giannoudis PV, Einhorn TA, Schmidmaier G, Marsh D (2008). The diamond concept—open questions. Injury.

[4] Klein-Nulend J, Bakker AD, Bacabac RG, Vatsa A, Weinbaum S (2013). Mechanosensation and transduction in osteocytes. Bone.

[5] Bailón-Plaza A, Van Der Meulen MCH (2003). Beneficial effects of moderate, early loading and adverse effects of delayed or excessive loading on bone healing. J Biomech.

[6] Goodship AE, Cunningham JL, Kenwright J (1998). Strain rate and timing of stimulation in mechanical modulation of fracture healing. Clin Orthop Relat Res.

[7] Yamaji T, Ando K, Wolf S, Augat P, Claes L (2001). The effect of micromovement on callus formation. J Orthop Sci.

[8] Claes LE, Heigele CA, Neidlinger-Wilke C (1998). Effects of mechanical factors on the fracture healing process. Clin Orthop Relat Res.

[9] Koval KJ, Sala DA, Kummer FJ, Zuckerman JD (1998). Postoperative weight-bearing after a fracture of the femoral neck or an intertrochanteric fracture. J Bone Joint Surg Am.

[10] Carrera I, Gelber PE, Chary G, González-Ballester MA, Monllau JC, Noailly J (2016). Fixation of a split fracture of the lateral tibial plateau with a locking screw plate instead of cannulated screws would allow early weight bearing: a computational exploration. Int Orthop.

[11] Gross SC, Galos DK, Taormina DP, Crespo A, Egol KA, Tejwani NC (2016). Can tibial shaft fractures bear weight after intramedullary nailing? A randomized controlled trial (Level of Evidence: Therapeutic Level I. See Instructions for Authors for a complete description of levels of evidence). J Orthop Trauma.

[12] Kazakia GJ, Tjong W, Nirody JA (2014). The influence of disuse on bone microstructure and mechanics assessed by HR-pQCT. Bone.

[13] Rezen T, Kovanda A, Eiken O, Mekjavic IB, Rogelj B (2014). Expression changes in human skeletal muscle miRNAs following 10 days of bed rest in young healthy males. Acta Physiol.

[14] Xia L, Cheung K-K, Yeung SS, Yeung EW (2016). The involvement of transient receptor potential canonical type 1 in skeletal muscle regrowth after unloading-induced atrophy. J Physiol.

[15] Müller ME, Koch P, Nazarian S, Schatzker J (1990). The comprehensive classification of fractures of long bones.

[16] Harwood PJ, Newman JB, Michael ALR (2010). (ii) An update on fracture healing and non-union. Orthop Trauma.

[17] Hustedt JW, Blizzard DJ, Baumgaertner MR, Leslie MP, Grauer JN (2012). Lower-extremity weight-bearing compliance is maintained over time after biofeedback training. Orthopedics.

[18] Ruiz FK, Fu MC, Bohl DD (2014). Patient compliance with postoperative lower extremity touch-down weight-bearing orders at a level I academic trauma center. Orthopedics.

[19] Whelan DB, Bhandari M, Stephen D (2010). Development of the radiographic union score for tibial fractures for the assessment of tibial fracture healing after intramedullary fixation. J Trauma Inj Infect Crit Care.

[20] Joslin CC, Eastaugh-Waring SJ, Hardy JRW, Cunningham JL (2008). Weight bearing after tibial fracture as a guide to healing. Clin Biomech.

[21] Blair JA, Stoops TK, Doarn MC (2016). Infection and nonunion after fasciotomy for compartment syndrome associated with tibia fractures. J Orthop Trauma.

[22] Metsemakers WJ, Roels N, Belmans A, Reynders P, Nijs S (2015). Risk factors for nonunion after intramedullary nailing of femoral shaft fractures: remaining controversies. Injury.

[23] Teraa M, Blokhuis TJ, Tang L, Leenen LPH (2013). Segmental tibial fractures: an infrequent but demanding injury. Clin Orthop Relat Res.

[24] Santolini E, West R, Giannoudis PV (2015). Risk factors for long bone fracture non-union: a stratification approach based on the level of the existing scientific evidence. Injury.

[25] Pountos I, Georgouli T, Blokhuis TJ, Pape HC, Giannoudis PV (2008). Pharmacological agents and impairment of fracture healing: what is the evidence?. Injury.

[26] Kurmis AP, Kurmis TP, O’Brien JX, Dalén T (2012). The effect of nonsteroidal anti-inflammatory drug administration on acute phase fracture-healing: a review. J Bone Joint Surg.

[27] Meling T, Harboe K, Enoksen CH, Aarflot M, Arthursson AJ, Søreide K (2012). How reliable and accurate is the AO/OTA comprehensive classification for adult long-bone fractures?. J Trauma Acute Care Surg.

[28] Gustilo RB, Anderson JT (2002). Prevention of infection in the treatment of one thousand and twenty-five open fractures of long bones. J Bone Joint Surg.

[29] Saatman KE, Duhaime A-C, Bullock R, Maas AIR, Valadka A, Manley GT (2008). Classification of traumatic brain injury for targeted therapies. J Neurotrauma.

[30] Westgeest J, Weber D, Dulai SK, Bergman JW, Buckley R, Beaupre LA (2016). Factors associated with development of nonunion or delayed healing after an open long bone fracture. J Orthop Trauma.

[31] Karhof S, Bastian OW, Van Olden GDJ, Leenen LPH, Kolkman KA, Blokhuis TJ (2017). Impaired fracture healing of the distal femur after high energy trauma. SM J Arthritis Res.

